# The Development of a Gas–Liquid Two-Phase Flow Sensor Applicable to CBM Wellbore Annulus

**DOI:** 10.3390/s16111943

**Published:** 2016-11-18

**Authors:** Chuan Wu, Guojun Wen, Lei Han, Xiaoming Wu

**Affiliations:** 1 Faculty of Engineering, China University of Geosciences (Wuhan), Wuhan 430074, China; wuchuancug@126.com (C.W.); weiliu181@126.com (X.W.); 2 Faculty of Mechanical and Electronic Information, China University of Geosciences (Wuhan), Wuhan 430074, China; hanleicug@126.com

**Keywords:** flow sensor, two-phase flow, wellbore annulus, CBM (Coalbed Methane)

## Abstract

The measurement of wellbore annulus gas–liquid two-phase flow in CBM (coalbed methane) wells is of great significance for reasonably developing gas drainage and extraction processes, estimating CBM output, judging the operating conditions of CBM wells and analyzing stratum conditions. Hence, a specially designed sensor is urgently needed for real-time measurement of gas–liquid two-phase flow in CBM wellbore annulus. Existing flow sensors fail to meet the requirements of the operating conditions of CBM wellbore annulus due to such factors as an inapplicable measurement principle, larger size, poor sealability, high installation accuracy, and higher requirements for fluid media. Therefore, based on the principle of a target flowmeter, this paper designs a new two-phase flow sensor that can identify and automatically calibrate different flow patterns of two-phase flows. Upon the successful development of the new flow sensor, lab and field tests were carried out, and the results show that the newly designed sensor, with a measurement accuracy of ±2.5%, can adapt to the operating conditions of CBM wells and is reliable for long-term work.

## 1. Introduction

China is rich in the reserve of CBM (coalbed methane). China’s CBM is buried under a depth of less than 2000 m with a volume of 36.81 trillion m^3^, accounting for approximately 15.3% of the world’s CBM reserve and ranking the third largest in the world [1,2]. To best exploit CBM resources, the Chinese government has accelerated the research and development of CBM extraction technologies in recent years and explored a set of CBM extraction techniques by drilling several test wells. However, vertical wells are commonly used in CBM extraction in China due to the limited technical conditions.

Due to the natural fracture structure of coalbeds, CBM wells need drainage and depressurization, during which groundwater and CBM are both produced from the vertical wellbore annulus, resulting in gas–liquid two-phase flow (hereinafter referred to as two-phase flow) in the wellbore annulus. For CBM wells, especially commingled CBM drainage and extraction wells, the measurement of wellbore annulus two-phase flow is of great significance for developing reasonable gas drainage and extraction processes, estimating CBM output, judging the operating conditions in CBM wells and analyzing stratum conditions [3]. Therefore, the real-time measurement of two-phase flow in CBM wellbore annulus is imperative.

Generally, the size of the wellbore annulus of common CBM extraction wells shall not exceed 26 mm, and the pressure of the operating environment is up to 10 MPa. Meanwhile, a few pulverized coal particles inevitably exist in the wellbore. Due to the above factors, the operating environment must be considered during the selection or design of the flow sensor.

Existing flow sensors can be divided into volumetric flow sensors and inferential flow sensors [4]. Due to their overlarge size, volumetric flow sensors cannot adapt to the narrow space of CBM wells. Inferential flow sensors include target flow sensors, differential pressure flow sensors, ultrasonic flow sensors, and so on, which are also inadequate for the operating environment of CBM wells. The detailed reasons are as follows.

(1) Target Flow Sensor [5]

Basic principles: When flowing through the target flow sensor, the fluid impacts the target bar. The impact force is transmitted from the target bar to the elastomer, causing the deformation of the elastomer. The deformation of the elastomer can be obtained from the deformation measurement component installed on the elastomer. The deformation amount is proportional to the flow.

Advantages: The target flow sensor is relatively simple in structure, not easily jammed, and convenient to maintain. It is adaptable to the environment with high viscosity, high contamination and suspended solid particles.

Disadvantages: The sensor causes large pressure loss.

Analysis: Existing target flow sensors are large, and their sealability fails to meet the requirement, so they are inapplicable to CBM wells.

(2) Differential Pressure Flow Sensor [6]

Basic principles: When fluids with different flow velocities flow through a pipeline with a variable cross-sectional diameter, a differential pressure will occur between the different cross-sections of the pipeline. The differential pressure is proportional to the flow velocities of the fluids. The differential pressure flow sensor is made according to this principle.

Advantages: The sensor is relatively simple in structure without moving parts, so it is highly reliable. With high linearity, it is widely applicable.

Disadvantages: The principle of the sensor determines that long, straight pipelines are needed at the front and back of the sensor, so its installation requirements are higher. In addition, it has disadvantages such as large size, narrow measurement range, large pressure loss, difficulty in measuring pipelines with small diameters, and large error.

Analysis: The differential pressure flow sensor is large due to the restriction of its measuring principle, and the CBM well environment fails to meet its installation requirements, so it cannot be used in CBM wells.

(3) Ultrasonic Flow Sensor [7]

Basic principles: When an ultrasonic wave passes through a fluid, the fluid flow will disturb the ultrasonic wave. Hence, the flow of the fluid can be obtained by comparing and analyzing the received ultrasonic signal and the original signal.

Advantages: The measurement is unaffected by the properties (electrical conductivity, temperature, pressure, viscosity, etc.) of the fluid. There is no contact between the sensor and the measured media, so the sensor is more suitable for the measurement of corrosive fluids. Its pressure loss is negligible and measurement accuracy is higher.

Disadvantages: The use and installation of the sensor are more complicated compared with common flow sensors. In addition, the sensor can be used only when the pipeline is filled with the fluid.

Analysis: The use and installation of the sensor are relatively complex, and the environment in CBM wells cannot meet its installation requirements. Meanwhile, the sensor requires the measured fluid to be a single medium or stable when measuring the flow, but the fluids (most two-phase flow and some pulverized coal particles) in a CBM wellbore annulus are not single media. Therefore, the sensor cannot be used in CBM wells.

(4) Electromagnetic Flow Sensor [8]

Basic principles: The electromagnetic flow sensor measures the fluid flow according to Faraday’s Electromagnetic Induction Law. When fluid flows through the sensor, the fluid will cut magnetic induction lines, which produce inductive electromotive force on both sides of the sensor electrode. The greater the flow, the greater the inductive electromotive force.

Advantages: The electromagnetic flow sensor is a noncontact flow measurement device, so its pressure loss is almost negligible when the fluid flows through the flow sensor, and its measurement accuracy is higher.

Disadvantages: With a higher requirement for the conductivity of the fluid, the sensor is inapplicable to fluids with low conductivity, such as steam and gas.

Analysis: With a higher requirement for measured fluids, the sensor is inapplicable to gas or two-phase flow, which has a higher void fraction, but the wellbore annulus two-phase flow is gas–liquid, and the void fraction is very high in annular flow and fine beam annular flow, so the sensor cannot be used in CBM wells.

(5) Floater Flow Sensor [9]

Basic principles: When the fluid enters the sensor from the inlet, differential pressure occurs between the upstream and downstream of the floater due to the closure effect of the floater. This differential pressure forces the floater to move along in the height direction of the sensor, eventually achieving a force balance state. Hence, the height of the floater is proportional to the flow of the fluid.

Advantages: The sensor has such advantages as simple structure, easy maintenance, low installation requirements, smaller and constant pressure loss, and wide measuring range.

Disadvantages: The sensor has low measurement accuracy. The measurement results are largely influenced by the viscosity, pressure, purity, density and temperature of the measured fluid.

Analysis: The measurement results of the sensor are susceptible to the physical parameters of the fluid, including viscosity, pressure, purity, density, and temperature. The above physical parameters of two-phase flow in a CBM wellbore annulus are all variable. Therefore, it cannot be used.

(6) Jet Flow Sensor [10,11]

Basic principles: Due to the special structure of the sensor, the Coanda effect occurs when the fluid enters the sensor. The additional feedback channel forces the fluid to produce oscillation inside the sensor. The oscillation frequency of the fluid is proportional to its flow. Therefore, the value of the flow can be obtained by measuring the oscillation frequency.

Advantages: The flow of the fluid is proportional to the oscillation frequency of the sensor. The measurement results are unaffected by the composition, pressure, temperature, viscosity, density, etc., of the fluid. With strong anti-interference capability and high stability, it is suitable for the measurement of medium- and high-speed flows.

Disadvantages: The flow sensor has such disadvantages as complex structure, large pressure drop, and large error during the measurement of low-speed flows.

Analysis: The sensor does not meet the requirement for the following reasons:

① This sensor is suitable for the measurement of medium- and high-speed flows, but the two-phase flow in the wellbore annulus is low-speed.
② Because of its complex structure and difficulty in processing, the sensor is difficult or even unable to be redesigned into a miniaturized form.

(7) Vortex Flow Sensor [12]

Basic principles: When viscous fluid at a certain velocity swirls around the vortex-generating body, the fluid micelle will form a pair of symmetric vortexes with inverse rotation directions somewhere behind the vortex-generating body, which is called the “Karman Vortex Street Phenomenon”. When the Reynolds number of the fluid is within a stable range, the vortex frequency is proportional to the flow. Therefore, the flow of the fluid can be obtained by measuring the vortex frequency by the sensor.

Advantages: The sensor has a wide scope of application (applicable to gas, liquid and steam), and the measurement results are unaffected by the composition, pressure, temperature, viscosity, density, etc., of the fluid. In addition, it has such advantages as simple structure, long service life, small pressure loss and high measurement accuracy.

Disadvantages: The pulsation and velocity distribution of the fluid exert a direct influence on the measurement accuracy. Moreover, the contamination of the vortex-generating body will also lead to large error in the measured results.

Analysis: During the use of this sensor, the pulsation of the fluid and the contamination of the vortex-generating body will exert great influence on the measurement accuracy of the sensor. Due to the obvious pulsation phenomenon of the two-phase flow in the wellbore annulus and the contamination of the vortex-generating body caused by pulverized coal particles, the sensor cannot be used.

(8) Turbine Flow Sensor [13]

Basic principles: When fluid flows through the sensor, the turbine blade of the sensor will rotate under the impetus of the fluid. The larger the flow, the greater the rotary speed. Therefore, the flow of the fluid can be obtained by measuring the rotary speed of the turbine blade.

Advantages: Among all flow sensors, the turbine flow sensor has the highest measurement accuracy. It also has other advantages such as a simple structure, strong anti-interference capability, and wide operating temperature range (−200 to 400 °C).

Disadvantages: The sensor has a high requirement for the measured medium; that is, the measured medium should be clean without particles. It is more suitable for fluids with high viscosity.

Analysis: The sensor has high cleanliness requirements for the measured medium, but the two-phase flow in the wellbore annular has poorer cleanliness. The bearing of turbine blades is easily jammed by pulverized coal particles, damaging the flow sensor. Therefore, the sensor cannot be used.

The above analysis shows that existing flow sensors cannot be used in a CBM wellbore annulus due to such factors as size, installation requirements, measuring principles, fluid media, and sealing performance. Among them, the target flow sensor can still be used under the conditions of high contamination and suspended solids and is suitable for the operating conditions of CBM wells. Meanwhile, based on its measurement principle, the sealing and size reduction of the sensor are easily realized. Therefore, it is finally determined that the new two-phase flow sensor be researched and developed based on the target flow sensor. Moreover, the two-phase flow pattern can be classified differently according to the gas content. Thus, the accuracy will increase greatly if the sensor can automatically discriminate and become calibrated to every flow pattern.

In conclusion, the basic principle of the sensor is that it can automatically discriminate and calibrate every flow pattern first and then automatically calibrate the flow value according to the deflection of the sensor’s part when impacted. The technological difficulties are as follows:

① The automatic discrimination and calibration of every flow pattern of the two-phase flow of CBM.
② The small size requirement, suitability of the sealing property for the conditions under the shaft, corrosion resistance, easy installation, suitability for the installation environment of the downhole tubing, etc.
③ The circuit to process the leak signal.

## 2. Measurement Indicators and Basic Principles

The measurement indicators must be determined before the design of the sensor, and the overall plan of the sensor should be put forward according to measurement indicators.

### 2.1. Measurement Indicators

According to the actual operating conditions of the CBM wellbore, the measurement indicators are summarized as follows:

(1) Sealability

The designed sensor can measure the two-phase flow in the CBM wellbore annulus. The designed applicable depth is 1000 m in the well, and the pressure of a 1000-m-deep waterspout is 10 MPa. Therefore, the designed sensor should have the sealability to withstand a water pressure of 10 MPa.

(2) Temperature

According to the geothermal gradient, the temperature increases by approximately 3 °C with each 100 m down from the earth’s surface [14], which means the temperature at the depth of 1000 m is nearly 30 °C more than that on the earth’s surface. Hence, the temperature tolerance range of the sensor is designed as 0–85 °C.

(3) Measurement Range

Through numerous investigations and research on the CBM wells in the field, the measurement range of the two-phase flow sensor is designed as 2–25 L/s.

(4) Accuracy

The accuracy of the sensor is ±2.5%, which is decided by the user of the project and the actual situation of achievable precision.

(5) Electric Parameters

To make the subsequent data acquisition circuit used in field of the project convenient, the output signal is a digital signal, and the supply voltage is 5 V.

To sum up, the basic design indicators of the flow sensor are as shown in Table 1.

### 2.2. Basic Principles

**Figure 1:**
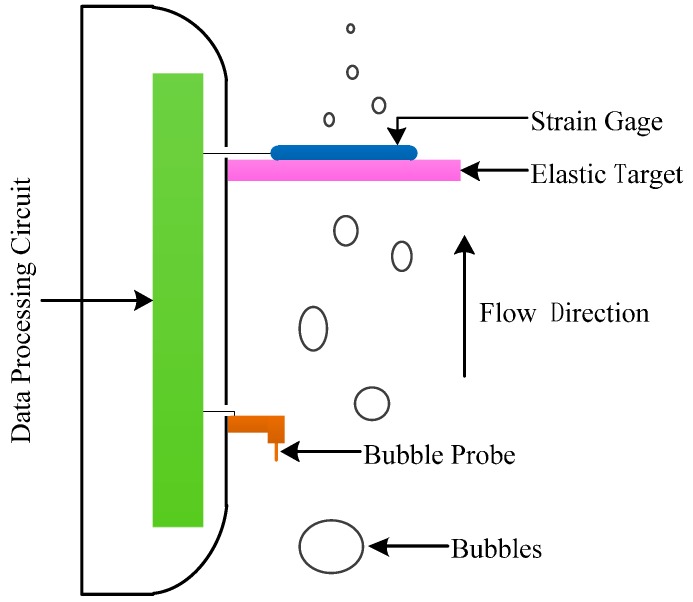
Basic principles of two-phase flow sensor.

According to the actual operating environment and measurement indicators, the basic principle diagram of the designed two-phase flow sensor is shown in Figure 1. When the two-phase flow impacts the elastic target along the direction shown in Figure 1, the elastic target is deformed by the impact force. The deformation is measured by the strain gage pasted on the elastic target. The greater the flow, the greater the deformation of the elastic target. Therefore, the specific value of the flow can be obtained through the calibration of the deformation signal output by the strain gage.

According to relevant theoretical knowledge, the gas–liquid two-phase flows can be subdivided into five patterns depending on the gas content: bubble flow, slug flow, churn flow, annular flow and fine beam annular flow [15]. In theory, when different patterns of two-phase flows impact the elastic target, the deformation of the elastic target is the same if the flows are the same. However, in fact, fluids with the same flow have different impacts on the elastic target due to their different densities, causing different deformations of the elastic target, thus resulting in error. Therefore, to reduce the error, the calibration of the two-phase flow sensor must be in accordance with different flow patterns of two-phase flows. Based on this, a bubble probe is added to the designed flow sensor for the measurement of two-phase flows (as shown in Figure 1). The calibration of flow signals is based on the detection results of flow patterns.

In conclusion, the data processing procedures (as shown in Figure 1) of the designed flow sensor are as follows:

(1) When passing through the sensor, the two-phase flow first passes through the probe. After the data processing circuit analyzes the collected bubble probe data, the flow pattern of the two-phase flow is obtained.
(2) As the two-phase flow moves on, the elastic target is deformed by the impact from the fluid. The deformation amount is measured by the strain gage, and the strain gage inputs the measured deformation signal into the data processing circuit.
(3) By matching the measured two-phase flow pattern to the corresponding calibration equation (each pattern of two-phase flow has its own calibration equation), the data processing circuit calibrates the deformation signal, finally obtaining the flow.
(4) The flow finally obtained by the data processing circuit is sent by the data cable.

## 3. Sensor Design

According to the foregoing discussion, the designed two-phase flow sensor consists of two parts, namely, bubble probe and elastic target, which will be introduced in the following content.

### 3.1. Bubble Probe Design

The bubble probe measures bubbles by detecting fluid conductivity. It judges the patterns of two-phase flows through the analysis of the quantity of the bubbles captured within a certain period and time information.

#### 3.1.1. Bubble Detection Principles

The bubble probe detects bubbles in the wellbore annulus based on the principle of liquid conductivity measurement. Both domestic and foreign scholars have carried out numerous studies on the conducting probe [16,17,18], and relevant theory and practice are mature. The bubble measurement principle of the bubble probe will be briefly introduced below.

**Figure 2:**
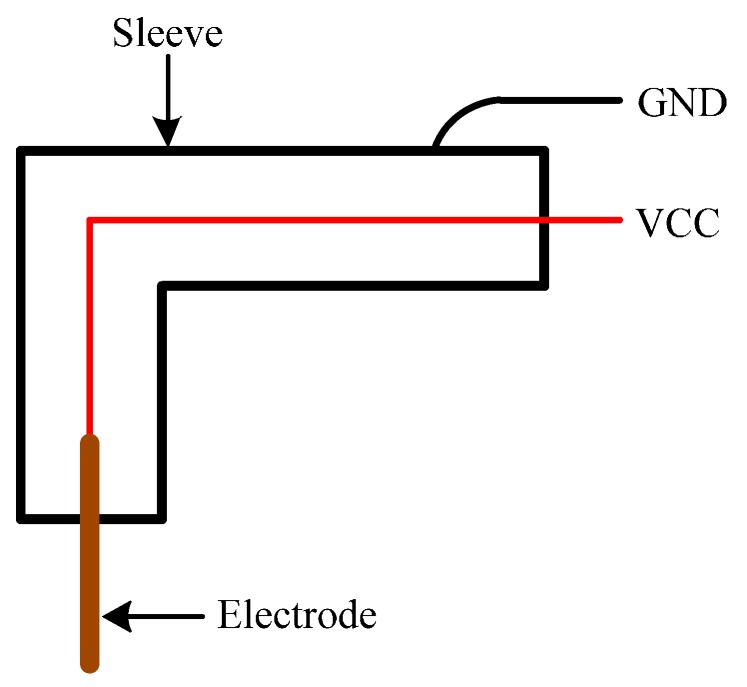
Structure principle diagram of bubble sensor.

The basic structure of the conducting probe designed in our sensor according to existing theory is shown in Figure 2. The probe is composed of an electrode and a sleeve, whose materials are both stainless steel. The electrode diameter is 0.3 mm, and the electrode is coated with insullac except at its end. The sleeve diameter should be as small as possible on the premise of wrapping the electrode and insulation from the electrode. The electrode is connected to VCC (the anode of the +5 V power supply), and the sleeve is connected to GND (the ground of the +5 V power supply). When the power supply is turned on, a conductivity test device is formed between the electrode and the sleeve.

**Figure 3:**
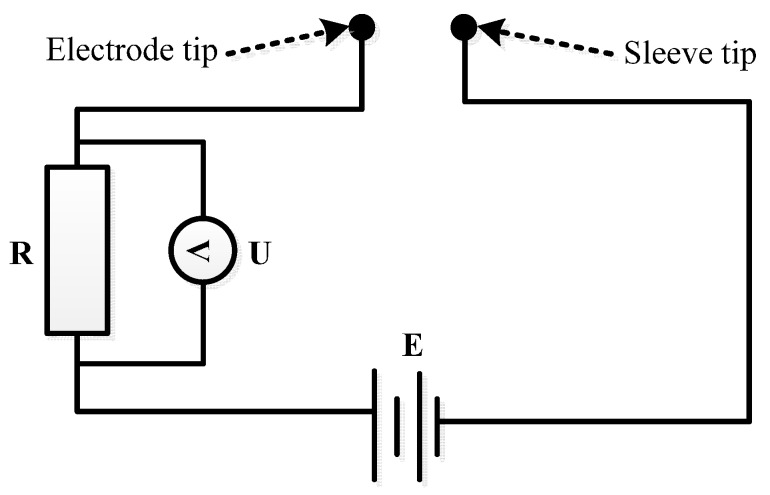
Circuit diagram of bubble probe.

The gas conductivity is 0, but the solution conductivity is not 0. Hence, the bubbles can be captured through detecting the significant differences between the conductibility of the two mediums. Through the simple circuit shown in Figure 3, the capture of bubbles can be realized. In Figure 3, *E* is power supply voltage, *R* is resistance (*R* = 3 kΩ), and *U* is the measured voltage between the two ends of *R*. The bubble detection principles are illustrated in Table 2.

Figure 3 and Table 2 show that when the solution connects the sleeve and the electrode, a closed circuit is formed because the solution conductivity is not 0, and the voltage *U* between the two ends of the resistance *R* is not 0. Conversely, when bubbles connect the sleeve and the electrode, an open circuit is formed because the gas conductivity is 0, and the voltage *U* between the two ends of the resistance *R* is 0. Due to the existence of interference signals and factors, the actual output signal when the bubbles pass through is not 0 but is very close to 0.

Therefore, the bubbles can be detected through the change of the voltage between the two ends of the resistance *R*; i.e., when the voltage *U* between the two ends of the resistance *R* is not 0, no bubble has passed through; when the voltage *U* between the two ends of the resistance *R* is 0, bubbles are passing through.

#### 3.1.2. Automatic Identification Principles of Two-Phase Flow

(1) Introduction of Two-phase Flow Patterns

**Figure 4:**
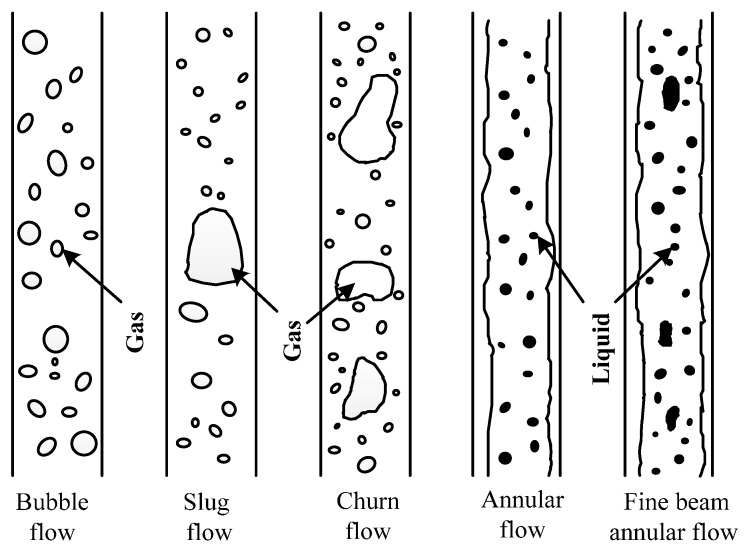
Pattern graph of two-phase flows in vertical ascending pipelines.

The flow patterns of two-phase flows can be classified according to different pipeline inclination angles. This paper studies the vertical CBM wells. Hence, for vertical ascending pipelines, with increasing gas content, the flow patterns of two-phase flows can be divided into bubble flow, slug flow, churn flow, annular flow and fine beam annular flow [15], as shown in Figure 4.

(2) Automatic Identification Principles of Two-phase Flow Patterns

Figure 4 shows that the patterns of two-phase flows are classified based on air content; namely, with increasing air content, the pattern of two-phase flow changes from bubble flow to fine beam annular flow successively. In appearance, as the bubble diameter of bubble flow increases gradually, the bubbles with larger diameters link together, finally forming a fine beam annular flow. Therefore, the pattern of two-phase flow can be roughly identified by judging the quantity of bubbles within a certain period. The method for pattern flow recognition aboveground and underground is to recognize and count the instances of high and low electrical levels [16]. This theory can also be used for the pattern flow’s automatic identification. Table 3 illustrates the automatic identification principles of two-phase flow, and the details are as follows:

① If several bubbles are detected during a fixed time 0–*t*_1_, it is a bubble flow.
② If only one bubble is detected during a fixed time 0–*t*_1_, and liquid phase occurs during a certain time *t*_1_–*t*_2_, then it is a slug flow or churn flow. Since slug flow and churn flow share common characteristics, they are classified into the same category.
③ If only one bubble is detected during a fixed time 0–*t*_3_, and the bubble is mixed with liquid phase within a short period, then it is an annular flow or fine beam annular flow. Since annular flow or fine beam annular flow share common characteristics, they are classified into the same category.
④ If only gas phase occurs during 0–*t*_3_, it is pure gas phase.
⑤ If only liquid phase occurs during 0–*t*_3_, it is pure liquid phase.

In Table 3, the value selection of *t*_1_, *t*_2_ and *t*_3_ is of great significance for flow pattern identification results. Unreasonable selection may lead to large error in flow pattern identification. The value determination of *t*_1_, *t*_2_ and *t*_3_ is related to the size of the designed flow sensor, the efficiency of the data processing chip, the sampling frequency and so on, so accurate calculation is impossible, and their values can be obtained only through many tests. The purchased two-phase flow simulator is used to carry out tests and can simulate the different patterns of the two-phase flow. We can obtain different output patterns of the two-phase flow by adjusting the values of *t*_1_, *t*_2_ and *t*_3_ of the sensor and then comparing it to the real pattern of the two-phase flow obtained by naked-eye observation. Finally, the error can be obtained when *t*_1_, *t*_2_ and *t*_3_ have different values. Some representative data are shown in Table 4.

We can draw the conclusion from Table 4 that, when the values of *t*_1_, *t*_2_ and *t*_3_ are too large or too small, the error will increase; thus, appropriate values are necessary. According to the test result, we decide to take *t*_1_ = 2.6 s, *t*_2_ = 3.9 s, and *t*_3_ = 4.5 s.

### 3.2. Elastic Target Design

When the same fluid impacts the elastic targets with different sizes, the greater the deformation of the elastic target, the more sensitive the designed sensor. Hence, the size of the elastic target should be accurately calculated to ensure the high sensitivity of the sensor. Meanwhile, the strain gage should be pasted on the greatest deformation point of the elastic target, which can improve the sensitivity of the sensor to the greatest extent. The calculation of the size of the elastic target and the position of the strain gage is given as follows.

**Figure 5:**
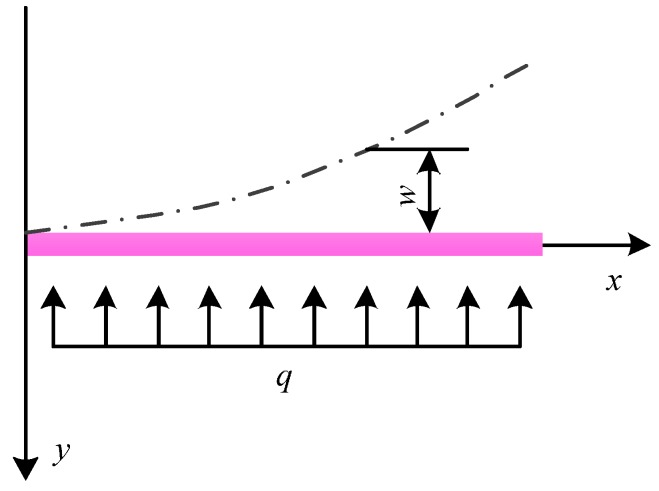
Physical model of elastic target under stress.

The physical model of the deformed elastic target under stress is shown in Figure 5. With the length direction of the elastic target as the *x*-axis (the elastic target is a slender axle, so its thickness can be ignored), and with the axial direction of the wellbore as the y-axis, a rectangular coordinate system is established. If the uniform load of the elastic target under the impact of the fluid is *q* and the bending deflection of the deformed elastic target under the impact of the load is *w*, then the deflection curve equation of the deformed elastic target is Equation (1):

(1) $$EIw=\int \left[\int M\left(x\right)dx\right]dx+c_{1}x+c_{2}$$

where *E* is the elastic modulus, *I* is the inertia moment of the cross section around the *z*-axis, the *z*-axis is perpendicular to the plane of the *x*-axis and *y*-axis, *w* is the deflection of any point on the *x*-axis, *M* (*x*) is the bending moment of any point on the *x*-axis, and *c*_1_ and *c*_2_ are unknown constants.

For the physical model shown in Figure 5, the deflection and angle at the fixed end (i.e., *x* = 0) are both 0; namely,

(2) $$\begin{array}{l} x=0\Rightarrow w=0\\ x=0\Rightarrow w^{\prime }=0 \end{array}$$

By substituting Equation (2) into Equation (1), the deflection curve equation of the deformed elastic target under stress can be obtained as Equation (3):

(3) $$w=\frac{x^{4}\cdot q}{12E\cdot I}$$

If the length of the elastic target is *a*, the width is *b*, and the thickness is *h*, then the calculation equation of the inertia moment *I* of the elastic target is Equation (4):

(4) $$I=\frac{h\cdot b^{3}}{12}$$

The only unknown parameter in Equation (3) is then uniform load *q*, and the calculation equation of *q* will be derived in the following part.

If the fluid quality impacting the elastic target during the time of ∆*t* is *m*, then

(5) m=v⋅Δt⋅A⋅ρ

where *m* is the fluid quality impacting the elastic target during ∆*t*, *v* is the velocity of the fluid, *A* is the impacted area of the elastic target, and *ρ* is the fluid density.

The impacted area *A* of the elastic target is then

(6) A=a⋅b

If the total impact force received by the elastic target during ∆*t* is *F*, then the below equation can be obtained according to the impulse theorem:

(7) F⋅Δt=Δm⋅v

According to the definition, the distributed load *q* is the force distribution in the length direction, and then

(8) $$q=\frac{F}{a}$$

The following equation can be obtained through the simultaneous Equations (5)–(8):

(9) $$q=b\cdot \rho \cdot v^{2}$$

By substituting Equations (4) and (9) into Equation (3), the deflection equation of the elastic target under stress can be obtained as given below:

(10) $$w=\frac{\rho \cdot v^{2}\cdot x^{4}}{E\cdot h\cdot b^{2}}$$

According to Equation (10), the elastic target length *a* (i.e., *x* in Equation (10)) is proportional to the deflection *w*, while the elastic target width *b* and height *h* are inversely proportional to the deflection *w*. In the practical application, greater deflection is better. The greater the deflection is, the greater the deformation and the more sensitive the corresponding sensor. Hence, during the size design of the elastic target, *a* should be designed as large as possible, and *b* and *h* should be as small as possible. Considering such factors as the spatial size of the wellbore annulus and the size of the sensor, and combining with many tests, it is determined that *a* = 18 mm, *b* = 6 mm, and *h*= 0.8 mm.

**Figure 6:**
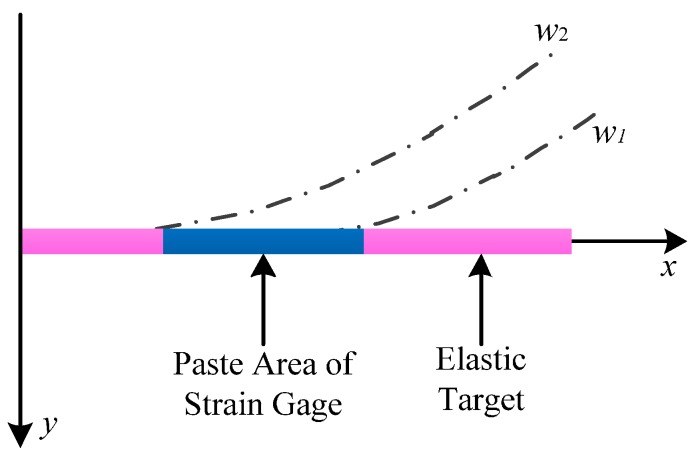
Paste area of strain gage.

When the size of the elastic target is determined, the only unknown parameter in Equation (10) is the velocity *v*. The velocity *v* is the ratio of the flow to the cross-sectional area of the pipeline where the sensor is. Hence, the velocity range can be obtained by dividing the flow range shown in Table 1 by the cross-sectional area of the pipeline. The deflection *w*_1_ of the elastic target can be obtained by substituting the minimal value of the velocity range into Equation (10), and the deflection *w*_2_ of the elastic target can be obtained by substituting the maximum value of the velocity range into Equation (10). The deflection curves *w*_1_ and *w*_2_ of the deformed elastic target under stress are shown in Figure 6. Figure 6 shows that the largest deformed region of the elastic target is the “root” between deflection curves *w*_1_ and *w*_2_, so the strain gage should be pasted on the “root”.

### 3.3. Connection Mode of Strain Gage

A full-bridge circuit is formed by connecting four same single-strain gages. When the resistance of any strain gage in the full-bridge circuit changes, the output voltage value of the full-bridge circuit will also be changed. Thus, the full-bridge strain gage is the most sensitive. Based on this, in the design of the two-phase flow sensor, four single-strain gages with the same resistance are connected to form a full-bridge circuit for measurement. When the elastic target is deformed, the output voltage *U* of the strain bridge is [19].

(11) $$U=\frac{kE\left(\varepsilon_{1}-\varepsilon_{2}+\varepsilon_{3}-\varepsilon_{4}\right)}{4}$$

where *k* is the strain gage constant, and *ε*_1_, *ε*_2_, *ε*_3_, and *ε*_4_ are the variable of the four strain gages.

### 3.4. Temperature Drift and Its Solution

Temperature drift occurs when making the sensor by strain gages. Therefore, specific causes for temperature drift of the sensor should be analyzed, and the solution for this problem should be proposed.

#### 3.4.1. Causes of Temperature Drift

In the case of no load, the output data of the sensor changes with the temperature—namely, temperature drift. When the temperature changes, theoretically, four strain gages with the same resistance in the full-bridge circuit change along with the temperature, and then the following equation is obtained:

(12) $$R_{1}+\Delta R=R_{2}+\Delta R=R_{3}+\Delta R=R_{4}+\Delta R$$

By substituting Equation (12) into Equation (11), the output voltage *U* of the full-bridge circuit remains the same, and temperature change exerts no influence on the output—namely, no temperature drift theoretically. However, in fact, many tests show that temperature drift still exists in full-bridge strain gages. The causes of temperature drift in the designed sensor are various, and the main causes are summarized as follows through experiments and theoretical analysis.

(1) Different Resistances of Strain Gages

**Figure 7:**
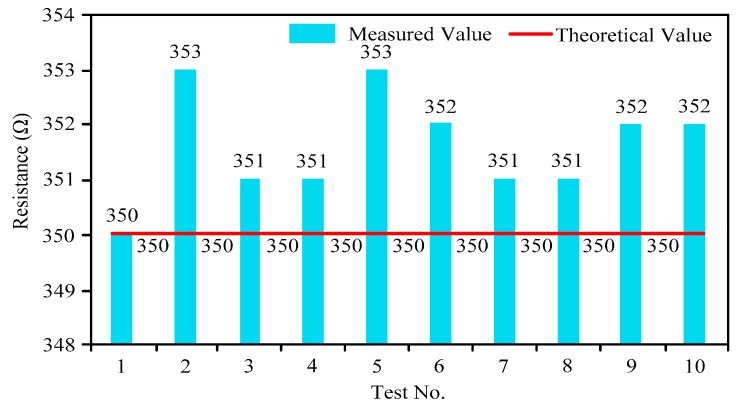
Actual resistance distribution of strain gages of the same specification.

According to the previous discussion and Equation (12), the full-bridge circuit must be established by several strain gages with the same resistance. However, due to such factors as production technology, the resistances of the strain gages cannot be identical. Figure 7 is the measured resistance distribution diagram of a batch of purchased strain gages (theoretical resistance is 350 Ω). Figure 7 shows that error exists between the measured values and the theoretical values of strain gages, so the strain gages with the same or similar resistances should be selected to establish the full-bridge circuit.

(2) Inhomogeneous Material of Elastic Target

Objects expand when heated and contract when cooled. Hence, if the material of the elastic target is inhomogeneous, the deformation of the elastic target is also uneven after being heated and cooled. Thus, the deformation amounts measured by the strain gage are different, finally causing temperature drift.

(3) Uneven Adhesive Coating

The strain gages are pasted to the surface of the elastic target by the adhesive. If the coating thickness of the adhesive is uneven or the adhesive is mixed with impurities, air bubbles, etc., the adhesive will be uneven. Moreover, if the temperature changes, the strain gage will dissipate heat unevenly due to the uneven coating of the adhesive, resulting in different deformations, finally causing temperature drift.

#### 3.4.2. Solution to Temperature Drift

A hardware circuit and software algorithm are combined to compensate for temperature drift.

(1) Hardware Compensation

**Figure 8:**
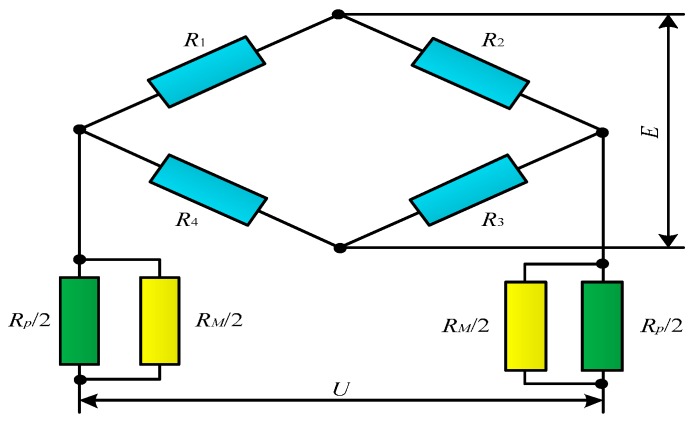
Hardware compensating circuit of strain gages.

In the hardware compensation method, the output end of the full-bridge circuit is connected in series with the resistance *R_M_* of high resistivity and high temperature coefficient [20]. When the temperature rises, the value of *R_M_* increases, and its partial pressure also increases. Since the power supply voltage *E* is constant, the power supply voltage of the bridge decreases. The output value of the full-bridge circuit will decrease with decreasing input voltage and rise with increasing temperature, so the output voltage can remain unchanged, solving the problem of temperature drift. However, when the full-bridge circuit is connected in series with the resistance *R_M_*, the linear relationship between the output voltage *U* and the deformation is destroyed. To solve this problem, the resistance *R_M_* should be connected in parallel with a metal film resistor *R_P_* of small temperature coefficient and high precision. The finally formed compensation circuit is as shown in Figure 8, in which *R*_1_, *R*_2_, *R*_3_ and *R*_4_ are strain gages of the same specification; *R_M_* is a resistance of high resistivity and high temperature coefficient; *R_P_* is a metal film resistor of small temperature coefficient; *E* is the power supply voltage; and *U* is the output voltage.

(2) Software Compensation

When the temperature increases, the output data of the sensor change. By analyzing the rules between the temperature and the output data of the sensor, the fitting curve equation between them is discovered. The output data of the sensor can be compensated according to the fitting curve equation. Because of the different production processes and sensor parameters, the fitting equations are also different. Thus, every sensor should be compensated by software after completion.

### 3.5. Zero Drift and Its Solutions

Zero drift exists in the designed flow sensor, so the specific causes for the occurrence of zero drift must be discovered and solved.

#### 3.5.1. Causes for Zero Drift

Under the condition of no load and constant temperature, the initial output data of the designed flow sensor should be 0 theoretically but in fact are not 0; i.e., zero drift occurs. The causes of zero drift are summarized as follows.

(1) Initial Deformation

In the fabrication process of the flow sensor, the strain gage has an initial deformation due to the unreasonable pasted position of the strain gage or the initial deformation of the elastic target, so the initial value of the sensor is not 0.

(2) Residual Deformation 

In the test process of the flow sensor, if the impact force of the fluid is too large, and an irreversible and permanent residual deformation may be caused on the elastic target, so the initial value of the sensor is not 0.

#### 3.5.2. Solutions to Zero Drift

**Figure 9:**
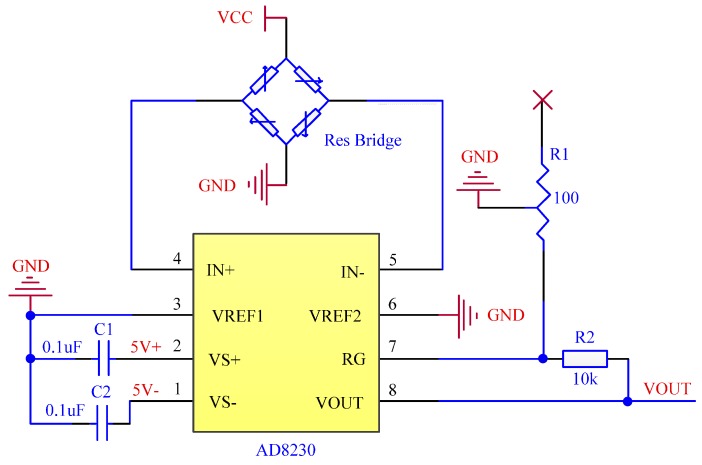
Circuit diagram of zero drift processing.

The hardware circuit is used to address the zero-drift problem of the sensor. The circuit is established with an AD8230 chip (AnalogDevices, Norwood, MA, USA). With automatic zero stabilization technology, the AD8230 chip can well solve the problem of zero drift. The circuit diagram of zero drift processing is shown in Figure 9.

The circuit shown in Figure 9 can not only deal with zero drift but also realize the functions of converting differential signals into single-ended signals and signal amplification, which are introduced as follows.

(1) Converting Differential Signals into Single-ended Signals

The built-in data processing chip of the sensor is used to process the input signal of the strain gage, but it can be used only to process the single-ended signal. The output signal of the full-bridge circuit is differential signal, so the circuit shown in Figure 9 should be used to convert the differential signal into the single-ended signal. The output end of the converted single-ended signal is the “VOUT” pin shown in Figure 9.

(2) Signal Amplification

The output voltage signal of the full-bridge circuit is very weak (mV level). Therefore, to increase the sensitivity of the sensor, the signal must be amplified. The circuit shown in Figure 9 can amplify the output signal of the full-bridge circuit, and the amplification times are determined by the resistance values of *R*_1_ and *R*_2_ in Figure 9; namely,

(13) G=2(1+R2R1)

where *G* stands for the amplification times.

After the completion of the full-bridge circuit by pasting, the amplification times of the circuit can be determined through tests. For the flow sensor designed in this paper, when the two-phase flow is small, the impact of the fluid on the elastic target is small, the elastic target is not deformed, and no signal is output from the elastic target. When the two-phase flow is greater than 2 L/s (the minimal value the sensor can detect), the signal begins to output from the elastic target. When the two-phase flow increases to 25 L/s (the maximum value the sensor can detect), the output signal of the elastic target reaches the maximum value of 10 mV.

In conclusion, within the measurement range of the sensor, the output signal of the elastic target is 0–10 mV. The built-in data processing chip of the sensor can collect the voltage signals of 0–5 V. Hence, to expand the sensitivity of the sensor, the output signal of the elastic target can be amplified from 0–10 mV to 0–5 V, i.e., 500 amplification times. In Equation (14), if *G* = 500, then *R*_1_ ≈ 40.16 Ω through calculation. Therefore, *R*_1_ is designed as the 0–100 Ω continuous adjustable precision potentiometer in the circuit, and 500 amplification times can be ensured by adjusting the resistance value of *R*_1_.

### 3.6. Sensor Calibration

**Figure 10:**
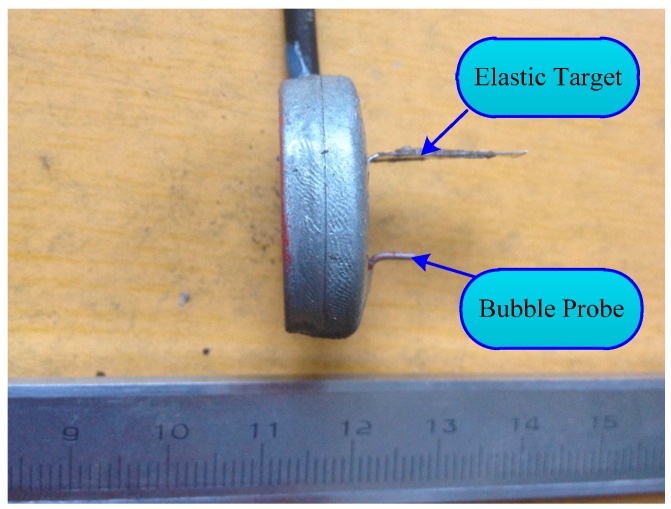
The two-phase flow sensor.

Given the above discussion, the designed flow sensor needs separate calibration for each pattern of two-phase flow. The completed sensor is shown in Figure 10. Sensor calibration should be based on many lab tests, and the test process is as follows.

(1) Test Device

The calibration is carried out on the purchased standard two-phase flow simulator, which can simulate different patterns of two-phase flows in the CBM wellbore annulus. With the data acquisition function, this device can measure and display the flow of the simulated two-phase flows in real time.

(2) Test Procedures

① Install the designed sensor on the two-phase flow simulator.
② Turn on the main power of the two-phase flow simulator, and the simulator will start to work.
③ Adjust the air inflow of the two-phase flow simulator to maintain the fluid in the simulator as bubble flow.
④ Open the data acquisition system of the simulator to record the two-phase flow.
⑤ Record the output voltage signals of the elastic target of the designed sensor.
⑥ Gradually adjust the two-phase flow of the simulator, and procedures ④ and ⑤ should be repeated for each adjustment.
⑦ Turn off the power supply of the whole system.

(3) Test Results

We can know that the deflection of the elastic target is proportional to the flow velocity from Equation (1) to Equation (10). Flow is the product of flow velocity and cross-sectional area, and the cross-sectional area of the CBM well field is a fixed value, so the deflection of the elastic target is proportional to the flow. Thus, we can calibrate the deflection of the elastic target into the flow.

**Figure 11:**
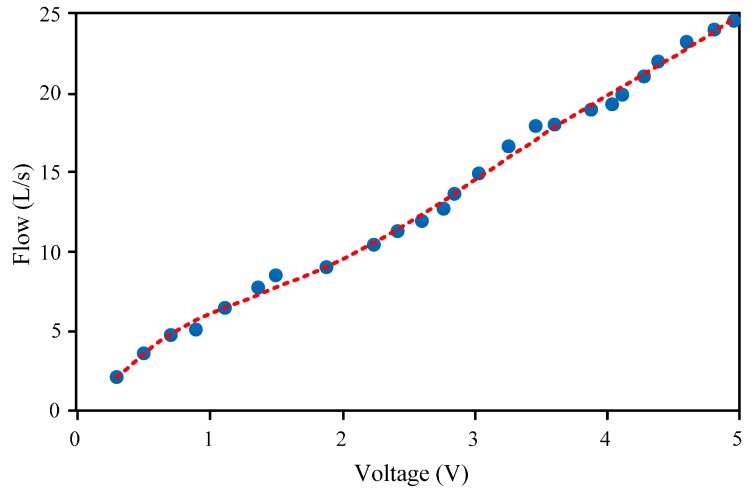
Calibration fitting curve of bubble flow.

The recorded output signal voltage values of the elastic target and the flow values of the simulator are matched one by one, obtaining the fitting curves, according to which calibration equations can be obtained. During the test, the fitting curve of bubble flow is shown in Figure 11, in which the abscissa is the output voltage signal value of the elastic target (unit: V), and the ordinate is the standard flow value measured by the corresponding simulator (unit: L/s).

The calibration equation of bubble flow can be fitted based on the data shown in Figure 11; i.e.,

(14) $$y=0.061x^{5}-0.8943x^{4}+4.798x^{3}-11.202x^{2}+15.057x-1.8528$$

where *y* is the flow of the fluid, *x* is the output signal voltage value of the elastic target, and 0.3 ≤ *x* ≤ 5.

**Figure 12:**
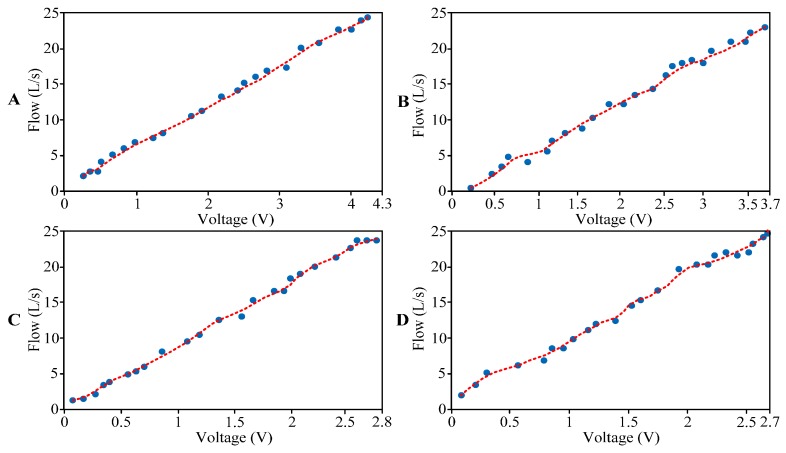
Calibration fitting curve of other flow patterns: (**A**) Slug flow; (**B**) Churn flow; (**C**) Annular flow; and (**D**) Fine beam annular flow.

Similarly, other patterns of two-phase flows are calibrated according to the above calibration process, and the fitting curve of other flow patterns are shown in Figure 12.

In conclusion, from Figure 11 and Figure 12, the calibration equations of different patterns of two-phase flows are shown in Table 5.

Table 5 shows that within the same measurement range, the values of *x* are different in different calibration equations. This phenomenon occurs because when fluids of the same flow but different densities impact the elastic target: the greater the fluid density, the greater the deformation amount of the elastic target—i.e., the greater the output voltage value. Thus, when the ranges of the two-phase flow are the same, the ranges of *x* in the above calibration equations decrease successively from bubble flow to fine beam annular flow.

Moreover, we can know that the deflection of the elastic target is proportional to the fluid density from the content shown earlier. Thus, the sensor was designed have the function to automatically discriminate and calibrate every flow pattern to improve the precision. However, when too many pulverized coal particles enter the two-phase flow, the average fluid density will be changed, which will have a certain influence on the test data. Thus, we should avoid pulverized coal particles.

## 4. Tests

Tests are divided into lab tests and field tests. Lab tests are aimed at testing the reliability and measurement error of the sensor, while field tests are aimed at testing the environmental adaptability of the sensor to the field operating conditions and its reliability for long-term work.

### 4.1. Lab Tests

The two-phase flow simulator is used to carry out the lab tests. Many experimental data are obtained by adjusting the different parameters of the two-phase flow simulator, and the measurement errors are obtained according to the experimental data. The sample frequency is 200 Hz. The number of tests for each flow pattern is 50, and the test time of each flow pattern is 10 min.

(1) Test Procedures

① Install the designed two-phase flow sensor on the two-phase flow simulator.
② Turn on the main power of the two-phase flow simulator, and the simulator will start to work.
③ Adjust the air inflow of the two-phase flow simulator to make the fluid in the simulator maintain the same pattern of two-phase flow.
④ Open the data acquisition system of the simulator to record the two-phase flow, and name this flow as standard flow.
⑤ Record the flow values output by the designed two-phase flow sensor, and name this flow as measured flow.
⑥ Constantly adjust the two-phase flow of the simulator, and record standard flow and measured flow.
⑦ Turn off the power supply to the whole system.

(2) Test Results

Some typical experimental data of bubble flow, slug flow, churn flow, annular flow and fine beam annular flow are shown in Table 6, Table 7, Table 8, Table 9 and Table 10.

The above tests and Table 6, Table 7, Table 8, Table 9 and Table 10 show that the measurement error of the designed flow sensor for bubble flow is ±2.5%. Similarly, many tests indicate that the measurement error of the designed flow sensor for other patterns of two-phase flows is also ±2.5%.

### 4.2. Field Tests

The field tests are aimed at testing the environmental adaptability of the sensor to the field operating conditions and its reliability for long-term work. The details of the field tests are as follows.

Test principles: Install the designed sensor to the specially made gauging nipple, and connect the gauging nipple to the field pipeline. By adjusting the pipeline length, the designed sensor can be placed into the well at the appointed depth. Meanwhile, the data collected by the sensor are transmitted to the ground terminal in real time for display and storage through cables. The sampling frequency of the sensor is 200 Hz, and the data memory card capacity of the ground terminal is 8 GB.

Test location: JS-064 Well, Lanyan CBM Co., Ltd., Jincheng City, Shanxi Province, China.

Test time: On 11 September 2015, the power supply was turned on to collect data. On 28 June 2016, the power supply was turned off to stop the data collection.

Test environment: During the tests, the temperature of the well bottom changed from 5 °C to 65 °C, and the pressure of the well bottom changed from 0 MPa to 10 MPa.

Test conclusions: The designed sensor worked stably and obtained reliable data, verifying that the designed sensor is adaptive to the operating conditions in CBM wells. In addition, nearly nine months of uninterrupted data collection verified the reliability of the designed two-phase flow sensor for long-term wok.

**Figure 13:**
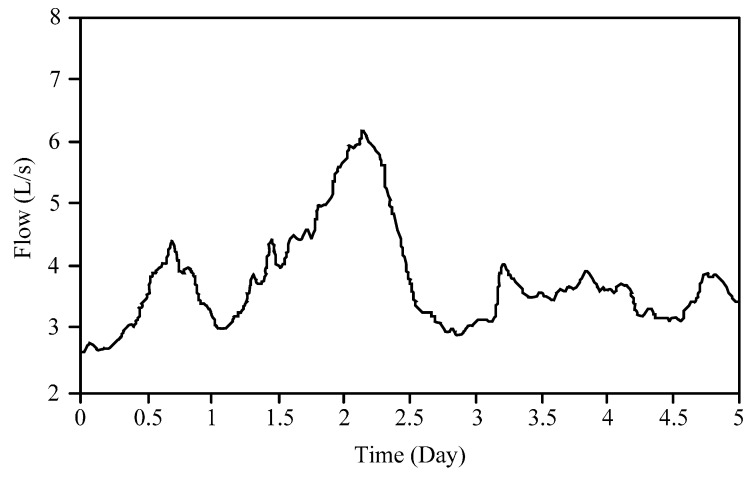
Curve of field test data during a period.

Figure 13 illustrates the curve of the field test data during a certain period. The abscissa is time (unit: day), and the ordinate is flow (unit: L/s). The following conclusions can be obtained by combining Figure 13 and the actual well site working conditions:

(1) On approximately Day 0.8, the first peak of two-phase flow occurred. The field operation data showed that the water of the wellbore output increased, but the gas output did not. Hence, the main cause for this peak was increasing water burst from the stratum, which was consistent with the actual field situation.
(2) On approximately Day 2.2, the second peak of two-phase flow occurred. The field operation data showed that the water of the wellbore output increased slightly, and the gas output also increased. Thus, the main cause of this peak was the increase of both the water burst from the stratum and the gas output, but the increasing gas output was the main cause, which was consistent with the actual field situation.
(3) From Day 2.5 to Day 5, the measured two-phase flow was relatively stable. The field operation data showed that the water of the wellbore output and the gas output were relatively stable, so the flow value was consistent with the actual field situation.

## 5. Conclusions

To measure the two-phase flow in a CBM wellbore annulus, a new two-phase flow sensor is designed based on the principle of a target flowmeter. The newly designed sensor can identify and automatically calibrate different two-phase flow patterns. Upon the successful development of the new flow sensor, lab and field tests were carried out, and the results show the following:

(1) The designed sensor, with a measurement accuracy of ±2.5%, is applicable to different patterns of two-phase flows.
(2) The designed sensor can adapt to the operating conditions of CBM wells (i.e., the annulus size is less than 26 mm, and the pressure is 10 MPa), and the long-term test results show that the reliability of the sensor is relatively high.
(3) Pulverized coal particles will slightly influence the measurement accuracy of the sensor. Therefore, the installation depth of the sensor should be reasonably arranged according to the geological data of the coalbeds, thus minimizing the contact between the sensor and pulverized coal particles.
(4) After the bubbles pass through the bubble probe on the sensor, the sensor can further calibrate the final flow, which indicates that the designed sensor is directional and can measure only unidirectional two-phase flows. Part of the CBM extraction well is vertical, and the gas–liquid two-phase flow is a vertical uplift; i.e., its flow direction is unidirectional. Thus, it can ensure the designed sensor’s operating requirement.

## Figures and Tables

**Table 1:** Calculation of design parameters of two-phase flow sensor.

Parameter	Value
Measurement Range	2–25 L/s
Measurement Error	±2.5%
Output Signal	Digital Signal Output
Supply Voltage	DC 5 V
Applicable Medium	Gas–liquid Two-phase Flow
Applicable Pressure	0–10 MPa
Applicable Temperature	0–85 °C

**Table 2:** Single bubble detection principles.

Medium	Solution	Bubbles
Open/Closed Circuit	Closed	Open
Output Voltage *U*	*U* ≠ 0	*U* = 0

**Table 3:** Automatic identification principles of two-phase flow patterns.

Patterns Time	*t*_1_	*t*_2_	*t*_3_
Bubble flow	Several bubbles	×	
Slug flow and churn flow	Only one bubble occurs	Liquid phase occurs	×
Annular flow and fine beam annular flow	Only one bubble occurs and it contains a small amount of liquid phase		This bubble still exists and contains a small amount of liquid phase
Gas phase	Pure gas		
Liquid phase	Pure liquid		

**Table 4:** The data of automatic discrimination of the two-phase flow pattern.

Test No.	*t*_1_		*t*_2_		*t*_3_	
	Value (s)	Error (%)	Value (s)	Error (%)	Value (s)	Error (%)
1	2.3	4.51	3.5	3.98	4.2	5.01
2	2.4	3.76	3.6	3.53	4.3	3.66
3	2.5	2.91	3.7	3.12	4.4	2.64
4	2.6	2.49	3.8	2.94	4.5	2.21
5	2.7	3.21	3.9	2.45	4.6	3.17
6	2.8	3.57	4.0	3.05	4.7	3.70
7	2.9	3.42	4.1	3.77	4.8	4.26
8	3.0	4.17	4.2	5.61	4.9	4.38

**Table 5:** Calibration equations of different patterns of two-phase flows.

Flow Patterns	Calibration Equations	Measurement Range
Bubble flow	$y=0.061x^{5}-0.8943x^{4}+4.798x^{3}-11.202x^{2}+15.057x-1.8528$ (0.3 ≤ x ≤ 5)	2–25 L/s
Slug flow	$y=-0.0343x^{5}+0.2856x^{4}-0.6598x^{3}+0.1386x^{2}+6.3614x+0.3876$ (0.26 ≤ x ≤ 4.25)	
Churn flow	$y=0.0186x^{5}-0.0901x^{4}-0.016x^{3}+0.368x^{2}+6.4952x+0.7141$ (0.19 ≤ x ≤ 3.69)	
Annular flow	$y=-0.228x^{5}+1.3736x^{4}-3.0203x^{3}+3.3119x^{2}+6.6887x+1.3399$ (0.11 ≤ x ≤ 2.78)	
Fine beam annular flow	$y=0.5966x^{5}-4.3646x^{4}+11.368x^{3}-12.438x^{2}+13.83x+0.7283$ (0.09 ≤ x ≤ 2.7)	

**Table 6:** Comparative analysis of measured data of bubble flow.

Test No.	Standard Flow (L/s)	Measured Flow (L/s)	Error (%)
1	2.03	2.08	+2.46
2	4.16	4.26	+2.4
3	6.59	6.45	−2.12
4	8.17	8.37	+2.45
5	10.02	9.89	−1.30
6	11.97	12.23	+2.17
7	14.24	13.91	−2.32
8	16.19	16.57	+2.35
9	17.86	18.21	+1.96
10	19.97	20.39	+2.10
11	22.68	23.23	+2.43
12	24.97	24.38	−2.36

**Table 7:** Comparative analysis of measured data of slug flow.

Test No.	Standard Flow (L/s)	Measured Flow (L/s)	Error (%)
1	2.07	2.12	2.42
2	3.92	3.87	−1.28
3	5.08	4.97	−2.17
4	7.35	7.43	1.09
5	10.72	10.48	−2.24
6	13.34	13.02	−2.4
7	15.06	15.42	2.4
8	17.91	17.64	−1.51
9	18.69	19.09	2.14
10	20.1	19.68	−2.09
11	23.58	23.07	−2.16
12	24.41	24.87	1.88

**Table 8:** Comparative analysis of measured data of churn flow.

Test No.	Standard Flow (L/s)	Measured Flow (L/s)	Error (%)
1	2.05	2.1	2.44
2	4.32	4.37	1.16
3	6.28	6.21	−1.11
4	7.1	7.27	2.4
5	9.97	10.18	2.11
6	12.24	12.45	1.72
7	15.37	15.06	−2.02
8	16.87	16.47	−2.37
9	18.91	19.31	2.12
10	21.02	20.65	−1.76
11	22.37	21.87	−2.24
12	24.07	24.56	2.04

**Table 9:** Comparative analysis of measured data of annular flow.

Test No.	Standard Flow (L/s)	Measured Flow (L/s)	Error (%)
1	2.13	2.18	2.35
2	3.96	4.03	1.77
3	5.65	5.58	−1.24
4	9.01	9.2	2.11
5	12.37	12.07	−2.43
6	13.48	13.27	−1.56
7	15.06	15.37	2.06
8	17.8	17.36	−2.47
9	19.42	19.83	2.11
10	21.08	21.57	2.32
11	23.2	22.64	−2.41
12	24.54	24.1	−1.79

**Table 10:** Comparative analysis of measured data of fine beam annular flow.

Test No.	Standard Flow (L/s)	Measured Flow (L/s)	Error (%)
1	2.17	2.22	2.3
2	4.05	4.15	2.47
3	6.23	6.11	−1.93
4	8.8	8.62	−2.05
5	10.16	10.27	1.08
6	12.57	12.26	−2.47
7	14.21	13.97	−1.69
8	16.7	17.05	2.1
9	18.26	18.61	1.92
10	20.09	20.5	2.04
11	22.13	22.67	2.44
12	24.32	24.9	2.38
